# Pyrrolyldihydropyrazino[1,2-a]indoletrione Analogue Microtubule Inhibitor Induces Cell-Cycle Arrest and Apoptosis in Colorectal Cancer Cells

**DOI:** 10.3390/molecules28041948

**Published:** 2023-02-17

**Authors:** Dong-Lin Yang, Hong-Xia Qin, Na-Na Zhang, Ya-Jun Zhang, Jiu-Hong Huang, Chun-Sheng Hu, Xiao-Xue Zhang, Yong Li, Liu-Jun He

**Affiliations:** 1 Chongqing Key Laboratory of Kinase Modulators as Innovative Medicine, College of Pharmacy, National & Local Joint Engineering Research Center of Targeted and Innovative Therapeutics, Chongqing University of Arts and Sciences, Chongqing 402160, China; 2 College of Pharmaceutical Sciences and Chinese Medicine, Southwest University, Chongqing 400715, China; 3 College of Biology and Food Engineering, Chongqing Three Gorges University, Chongqing 404020, China

**Keywords:** DHPITO, colorectal cancer cells, tubulin, G2/M phase, apoptosis, cell migration

## Abstract

In this study, 2-benzyl-10a-(1*H*-pyrrol-2-yl)-2,3-dihydropyrazino[1,2-*a*]indole-1,4,10(10a*H*)-trione (DHPITO), a previously identified inhibitor against hepatocellular carcinoma cells, is shown to exert its cytotoxic effects by suppressing the proliferation and growth of CRC cells. An investigation of its molecular mechanism confirmed that the cytotoxic activity of DHPITO is mediated through the targeting of microtubules with the promotion of subsequent microtubule polymerisation. With its microtubule-stabilising ability, DHPITO also consistently arrested the cell cycle of the CRC cells at the G2/M phase by promoting the phosphorylation of histone 3 and the accumulation of EB1 at the cell equator, reduced the levels of CRC cell migration and invasion, and induced cellular apoptosis. Furthermore, the compound could suppress both tumour size and tumour weight in a CRC xenograft model without any obvious side effects. Taken together, the findings of the present study reveal the antiproliferative and antitumour mechanisms through which DHPITO exerts its activity, indicating its potential as a putative chemotherapeutic agent and lead compound with a novel structure.

## 1. Introduction

Colorectal cancer (CRC) is globally one of the most prevalent malignant tumours, and is associated with high rate and mortality rates [1]. It is estimated that the number of cases of CRC is increasing annually, and this type of cancer poses a serious threat to human life and health as a result of economic development and lifestyle changes. Although the available therapeutic options for treating CRC have improved greatly over the last few decades, the prognosis for patients diagnosed with advanced-stage CRC remains dismal [1,2]. Blocking mitotic progression by targeting the microtubules of proliferating tumour cells is a consolidated strategy, and mitotic inhibitors are widely used in combination treatments for various types of tumours [3]. Therefore, the development of novel tubulin inhibitors is urgently required in order to offer more effective treatments for patients with CRC and other tumour types.

Microtubules are composed of α- and β-tubulin heterodimers assembled into linear protofilaments, and their packing involves lateral and longitudinal interactions between tubulins [4]. It is well-established that microtubules fulfil a crucial role in the cytoskeleton and are associated with several cellular processes, including organelle and vesicle transport, cell signalling, protein trafficking, and cell motility, division, and mitosis [5]. Microtubules form highly dynamic spindles during mitosis that are involved in the process of chromosome segregation into two daughter cells. The perturbation of microtubule dynamics may lead to cell-cycle arrest at the G2/M phase, and the formation of abnormal mitotic spindles eventually causing the induction of apoptosis [6]. Considering that microtubules participate in essential functions in cell motility and mitosis, they are highly attractive as a potential target for the development of anticancer drugs.

Depending on their mechanism of action, microtubule-targeting agents can be essentially divided into two categories: Microtubule-depolymerising agents and microtubule-polymerising agents [7]. Microtubule-targeting agents, as used in cancer therapy, through misdirecting the formation of the mitotic spindle in proliferating cancer cells, either cause mitotic arrest or lead cells into apoptosis. Microtubule-destabilising agents, such as vinblastine and colchicine, affect spindle formation and inhibit microtubule polymerisation, whereas microtubule-stabilising agents, such as paclitaxel and docetaxel, stimulate microtubular polymerisation [8,9]. As disrupting microtubule dynamics inhibits the growth of tumour cells, the development of novel microtubule-targeting chemotherapeutic drugs has attracted much attention.

In our previous study, a diverse suite of 2-disubstituted indolin-3-one and indole-3-one fused pyrazinos were constructed using a unique Ugi/Dieckmann condensation protocol [10]. Moreover, some of these synthesised compounds were identified as inhibitors against hepatocellular carcinoma cells. However, the underlying mechanism through which these compounds are able to mediate their inhibitory capabilities in hepatocellular carcinoma cells and their effects on other types of cancer cells, are yet to be fully elucidated. Furthermore, it remains unclear whether DHPITO could exert antitumour activity against colorectal cancer (CRC) cells and what the precise underlying mechanism for its antiproliferative action could be. In order to address these issues, the present study investigates the antitumour activity of DHPITO against CRC cells both in vivo and in vitro, and elucidates the underlying molecular mechanism. 

## 2. Results

### 2.1. DHPITO Exerts Cytotoxic Effects against CRC Cells In Vitro

**Figure 1:**
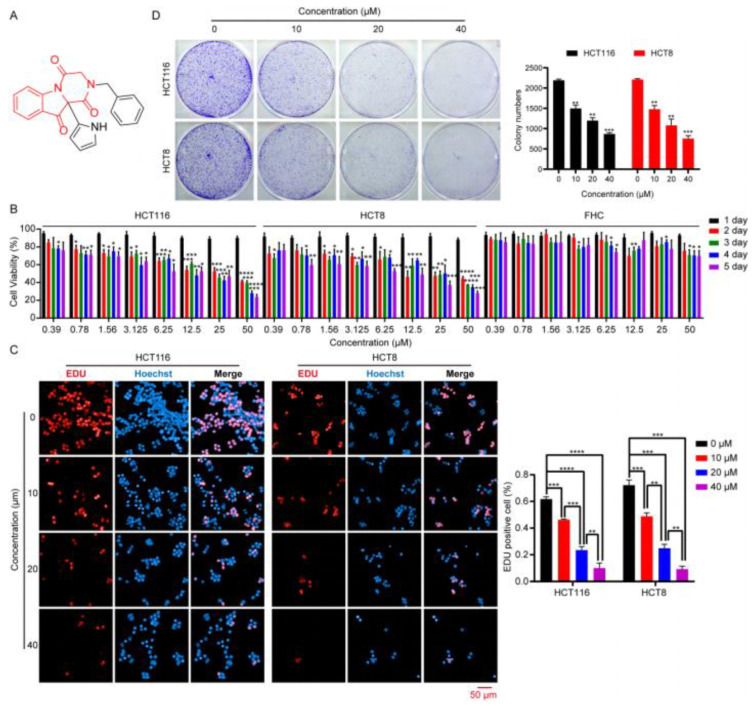
DHPITO suppresses the proliferation and growth in CRC cells. (**A**) Chemical structure of DHPITO. (**B**) HCT116, HCT8, and normal human rectal mucosal cells (FHC cell line) treated with the indicated concentrations of DHPITO for 1, 2, 3, 4, or 5 days. Relative cell viability was measured with an MTT assay. (**C**) EdU-staining assay was performed to further investigate the proliferative inhibition after treatment with DHPITO for 48 h. EdU-positive cells were counted and compared with the vehicle control group. Scale bar, 50 μm. (**D**) Colony formation assay was performed to evaluate growth in vitro after treatment with DMSO and 10, 20, or 40 μM DHPITO for 10 days. The colonies were visualised with crystal violet staining. All data are shown as the mean ± SD for three independent experiments. Ns, no significance. * *p* < 0.05; ** *p* < 0.01; *** and **** *p* < 0.001 versus vehicle. EdU, 5-ethynyl-2’-deoxyuridine; CRC, colorectal carcinoma.

Our research group previously designed and synthesised a series of compounds targeting tubulin, and found that these compounds were able to exert significant inhibitory activity against hepatocellular carcinoma cells [10]. In order to evaluate the therapeutic potential of these derivatives in CRC cells, an MTT assay was employed to assess their cytotoxicity in HCT116 cells. Interestingly, among all the tested compounds, DHPITO exhibited considerable inhibitory activity against HCT116 cells (Figure S1). The chemical structure of DHPITO is shown in Figure 1A. DHPITO caused a marked decrease in the cell viability of the HCT8 and HCT116 cell lines in both a dose- and a time-dependent manner (Figure 1B and Figure S2). By contrast, only mild cytotoxicity was associated with exposure to DHPITO in normal adult colonic epithelial cell line FHC (Figure 1B and Figure S2). Subsequently, EdU and colony formation assays were performed to determine the inhibitory effects of DHPITO on the proliferation and growth of CRC cells. As shown in Figure 1C, the rate of incorporation of thymidine analogue EdU into DNA declined with DHPITO exposure, and this decline occurred in a dose- and time-dependent manner. Consistently, both colony number and size were significantly reduced in a dose-dependent manner following the exposure to DHPITO (Figure 1D), whereas only a mild inhibitory growth effect was observed in normal FHC cells (Figure S3), suggesting that DHPITO could inhibit the proliferation and growth of the CRC cells. 

### 2.2. DHPITO Promotes Cellular Tubulin Polymerisation 

**Figure 2:**
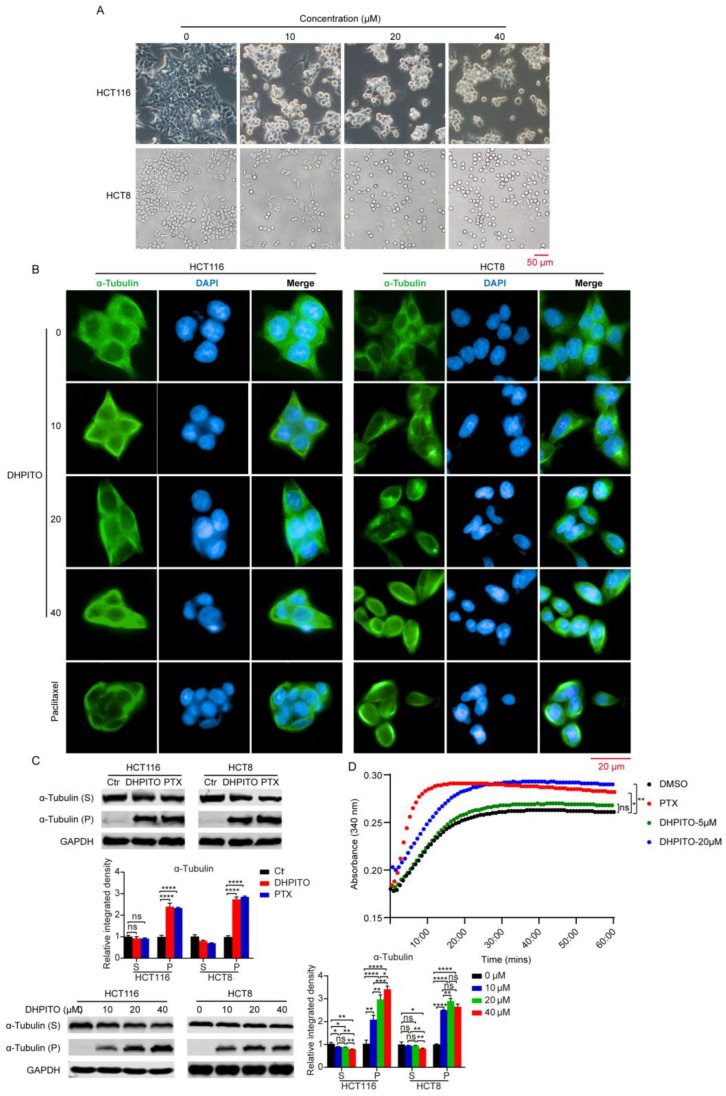
DHPITO acts as a microtubular polymerisation stabiliser. (**A**) Cell morphology of HCT116 and HCT8 cells was observed under a phase-contrast microscope in the presence of DHPITO. Scale bar: 50 μm. (**B**) Immunofluorescence staining showing that microtubular networks were stabilised after exposure to DHPITO or paclitaxel compared with the control cells. Microtubules were recognised with an anti-α-tubulin antibody (green), and nuclei were stained with DAPI (blue). Images were captured with fluorescence microscopy. Scale bar: 20 μm. (**C**) Enhancement of tubulin polymerisation in live cells. HCT116 and HCT8 cells were treated with paclitaxel (500 nM) and DHPITO (40 μM) for 24 h. Cells then were lysed and fractionated from the cytosol (supernatant, S) to cytoskeletal (pellet, P) extracts. The extracts were subjected to Western blotting using anti-α-tubulin and anti-GAPDH antibodies. ‘S’, unassembled tubulin; ‘P’, assembled tubulin. The detection of tubulin polymerisation is based on the increase in tubulin in the pellets, and its decrease in the supernatant. (**D**) Effects of DHPITO at 5 and 20 µM on the in vitro polymerisation of purified tubulin. Purified tubulin was polymerised in a cell-free assay. Tubulin protein was incubated at 37 °C in the absence (vehicle control) or presence of paclitaxel and DHPITO. Absorbance at 340 nm was monitored every 1 min for 60 min. Ns, no significance. * *p* < 0.05; ** *p* < 0.01; *** and **** *p* < 0.001 versus vehicle. DAPI, DAPI (4’,6-diamidino-2-phenylindole,2-(4-amidinophenyl)-1H-indole-6-carboxamidine.

Given that these compounds targeting tubulin were designed and synthesised in our previous study [10], we considered whether DHPITO was a bona fide inhibitor of tubulin. As anticipated, morphological observations revealed that the HCT116, HCT8, and SW480 cells grew in round grapelike clusters following DHPITO treatment, and these morphological changes were very similar to those observed in cells treated with paclitaxel, which is a microtubule depolymerisation inhibitor that binds to the tubulin polymer (Figure 2A and Figure S4). These findings were further confirmed with immunofluorescence analysis using an α-tubulin antibody, which revealed that DHPITO- and paclitaxel-treated cells possessed a much higher fluorescence intensity, attributable to α-tubulin in the cell membrane when compared with the negative control cells (Figure 2B). Similarly, treatment with both paclitaxel and DHPITO induced tubulin polymerisation, as evidenced by an increase in the cytoskeletal-tubulin (pellet, P) fraction and a concomitant decrease in the cytosolic-tubulin (S) fraction. Moreover, the exposure of HCT116 and HCT8 cells to DHPITO led to an increase in the amount of tubulin in the P fraction in a dose-dependent manner (Figure 2C). To further investigate whether tubulin was a target of DHPITO and its effects on the assembly kinetics of tubulin in vitro, an in vitro tubulin polymerisation assay using purified tubulin was performed with paclitaxel as a positive control. These experiments revealed that, compared with the DMSO control, DHPITO could enhance tubulin polymerisation in a dose-dependent manner. Under similar conditions, paclitaxel also promoted the assembly of tubulin, as shown in Figure 2D. Taken together, these data suggest that DHPITO may be an inhibitor of tubulin that is able to promote and stabilise microtubular polymerisation.

### 2.3. DHPITO Induces G2/M Phase Cell-Cycle Arrest

**Figure 3:**
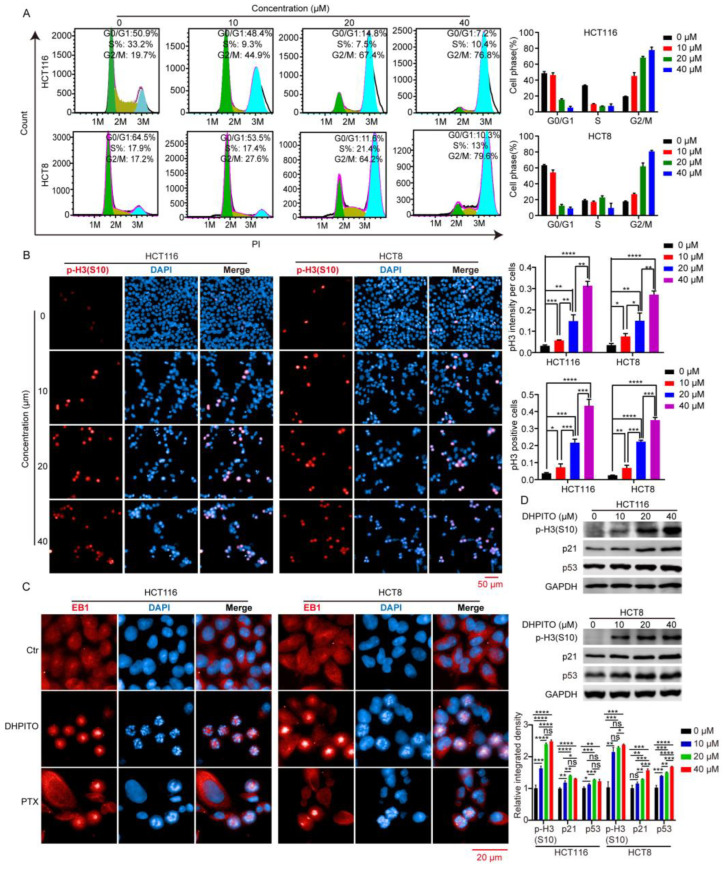
DHPITO induces cell-cycle arrest at G2/M phase. (**A**) Cell-cycle distribution of HCT116 and HCT8 cells analysed via flow cytometry following treatment with DHPITO for 48 h. DMSO was used as the vehicle control group. Histograms represent percentages of cell distribution in the G0/G1-, S- and G2/M-phases. Data are shown as the means ± SD (*n* = 3). (**B**) Mitotic cell-cycle marker P-H3 at Ser10 was measured with immunofluorescence after exposure to the indicated concentrations of DHPITO. Cells stained with a specific antibody (red) against p-H3 (Ser10) and nuclei stained with DAPI (blue) were visualised. (**C**) Effect of DHPITO on expression level of G2/M phase-associated proteins. P-H3 (Ser10), p21, and p53 in HCT116 and HCT8 cells were detected with Western blotting. GAPDH was used as a loading control. Scale bar, 50 μm. (**D**) Immunofluorescence colocalisation between EB1 and chromosomes was performed to evaluate the effect of DHPITO on chromosomal separation. Cells stained with a specific antibody (red) against EB1 and DAPI (blue) were captured. Scale bar: 20 μm. Ns, no significance. Data values are shown as the mean ± SD (*n* = 3). * *p* < 0.05; ** *p* < 0.01; *** and **** *p* < 0.0001. P-H3, phosphor-histone H3; DAPI, DAPI (4’,6-diamidino-2-phenylindole,2-(4-amidinophenyl)-1H-indole-6-carboxamidine; EB1, microtubular end-binding protein 1.

Due to the essential role that tubulin plays in mitosis, DHPITO may effectively block the progress of mitosis. As anticipated, the flow cytometric results revealed that DHPITO dose-dependently led to a significant increase in the number of cells at the G2/M phase (Figure 3A). To further evaluate its influence on progression of mitosis, mitotic cell-cycle marker phosphor-histone 3 (p-H3) at Ser 10 was measured using immunofluorescence analysis. These experiments show that an increase in the concentration of DHPITO that was used to treat the cells led to a concomitant marked increase in the fluorescence intensity, attributable to the nuclear p-H3 levels when compared with cells treated with solvent only (Figure 3B). Consistent with the observed increase in the G2/M-phase cell population, the levels of p-H3, p21, and p53 were increased following treatment with DHPITO for 48 h (Figure 3C). Given that microtubular protein EB1 localises to microtubular plus ends to regulate cell division [11], we performed experiments to examine the immunofluorescence colocalisation of EB1 with chromosomes in order to evaluate the effect of DHPITO on chromosomal separation. As shown in Figure 3D, similarly to paclitaxel, DHPITO led to a notable suppression of the normal chromosomal movement to both poles (as indicated by the blue signal in the figure), and the accumulation of EB1 (as denoted by the red signal) at the cell equator was promoted. Similarly, immunoblotting analysis demonstrated that DHPITO was able to promote EB1 accumulation in a dose-dependent manner (Figure S5), further confirming the effect of DHPITO on chromosomal movement. Taken together, these results suggest that the failure of microtubular depolymerisation in the presence of tubulin inhibitors triggered an aggregation of microtubules and chromosomes at the equatorial plate that subsequently resulted in chromosomes or chromosomal fragments remaining between the two poles. 

### 2.4. DHPITO Triggers Apoptosis in Both HCT116 and HCT8 Cells

**Figure 4:**
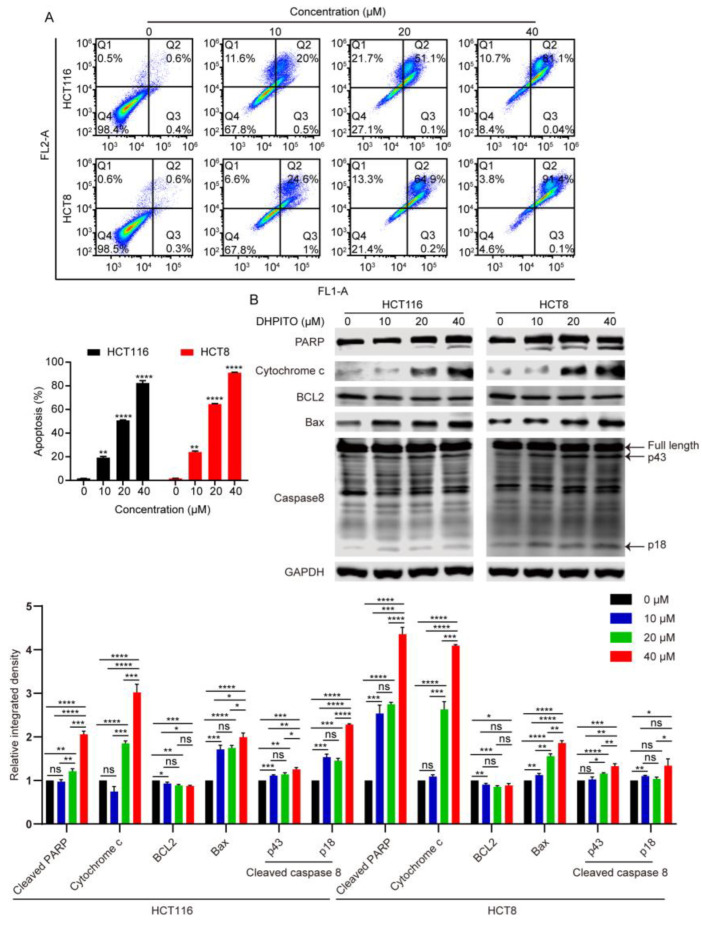
DHPITO activates apoptosis in both HCT116 and HCT8 cell lines. (**A**) Flow cytometric analysis using annexin V/PI staining indicates that cell apoptosis was induced by DHPITO in HCT116 and HCT8 cells. (**B**) Protein levels of cleaved PARP, cytochrome c, BCL2, BAX, and cleaved-caspase 8 detected with Western blotting following treatment with the indicated concentrations of DHPITO for 24 h. GAPDH was used as a loading control. Ns, no significance. * *p* < 0.05; ** *p* < 0.01; *** and **** *p* < 0.001 versus vehicle. PI, propidium iodide; PARP, poly ADP-ribose polymerase; BCL2, B-cell lymphoma 2; BAX, BCL2 -associated X protein.

It is generally accepted that mitotic catastrophe ultimately causes apoptosis [12]. In this series of experiments, our aim was to establish whether DHPITO could induce apoptosis via regulating the progress of mitosis. Consequently, flow cytometric analysis indicated that DHPITO led to a significant induction of apoptosis in both HCT116 and HCT8 cells, and the proportions of cells in late-phase apoptosis were increased in a dose-dependent manner (from 0.1 to 81.1% for HCT116 cells (*p* < 0.001), and from 0.6 to 91.4% for HCT8 cells (*p* < 0.001)) (Figure 4A), whereas this effect of promoting apoptosis was not observed in FHC cells (Figure S6). Furthermore, Western blot analysis reveals that DHPITO treatment triggered notable increases in the levels of cleaved PARP, cytochrome *c,* and pro-apoptotic proteins BAX and cleaved caspase-8, whereas the expression level of antiapoptotic protein BCL2 was decreased in a dose-dependent manner (Figure 4B). Taken together, these data indicate that DHPITO could induce apoptosis in CRC cells through the induction of G2/M arrest and mitotic catastrophe.

### 2.5. DHPITO Dose-Dependently Inhibits Cell Migration and Invasion 

**Figure 5:**
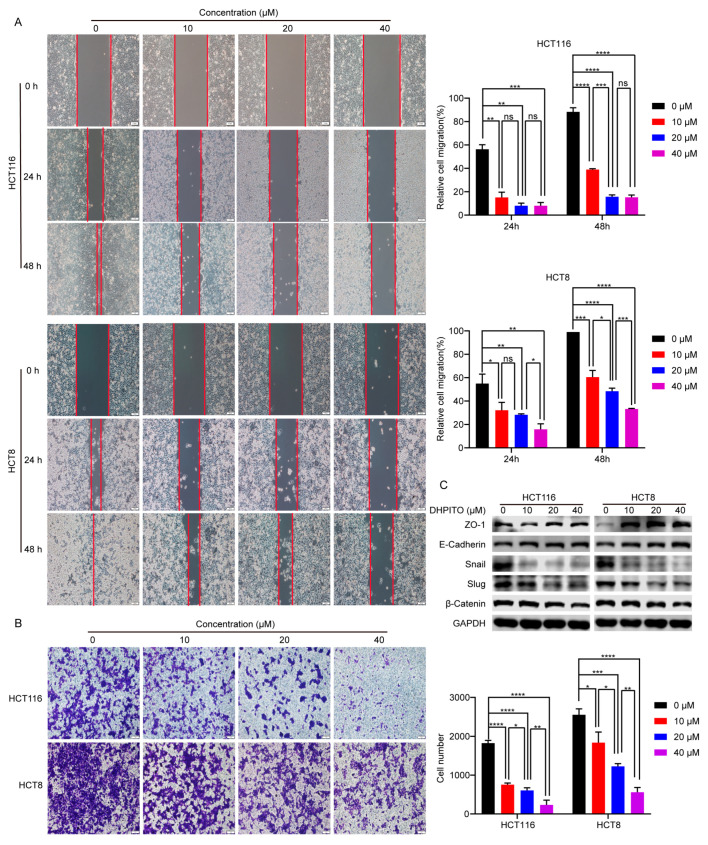
DHPITO reduces the motility of CRC cells. (**A**) Wound-healing assay was performed in 6-well plates with HCT116 and HCT8 cells, and drug solutions of different concentrations of DHPITO were incubated with the cells for 48 h to evaluate the effect of the compound. The inhibition of cell migration mediated by DHPITO was determined by comparing the wound closure. (**B**) Cell invasion was determined with a Transwell invasion assay. Representative images are shown at 40 × magnification. (**C**) The expression levels of epithelial markers (E-cadherin, ZO-1, β-catenin), mesenchymal markers (Snail and Slug), and β-catenin were investigated to confirm the effect of DHPITO on the motility of CRC cells. GAPDH was used as a loading control. All data are shown as the mean ± SD for three independent experiments. * *p* < 0.05; ** *p* < 0.01; *** and **** *p* < 0.00; ns, no significant difference versus vehicle. CRC, colorectal carcinoma.

Microtubules fulfil a key role in the motility, migration, and invasion of tumour cells through the assembly and disassembly of the α-tubulin and β-tubulin complexes [13]. To investigate the effects of DHPITO on the migratory potential of CRC cells, a scratch-wound assay was performed to detect the extent of cell migration. As shown in Figure 5A, wound-healing assay revealed that DHPITO could significantly impair the migration capability of CRC cells in a dose-dependent manner compared with the vehicle control. Subsequently, we sought to further explore whether DHPITO exerted any role in regulating cell motility of CRC cells; therefore, Transwell migration assays were performed. These experiments showed that treatment with DHPITO led to a marked reduction in the ability of CRC cells to penetrate the Matrigel-coated membrane (Figure 5B), suggesting that DHPITO could dose-dependently inhibit cell invasion. Consistently, Western blot analysis showed that treatment with DHPITO led to a significant increase in the expression levels of epithelial markers (E-cadherin, zona occludin-1 (ZO-1) and β-catenin), whereas the expression levels of mesenchymal markers (Snail and Slug) and β-catenin were reduced (Figure 5C). Collectively, these findings suggest that DHPITO was able to suppress not only tumour cell migration, but also the invasive motility of CRC cells. 

### 2.6. DHPITO Exhibits Strong Antitumour Activity In Vivo

**Figure 6:**
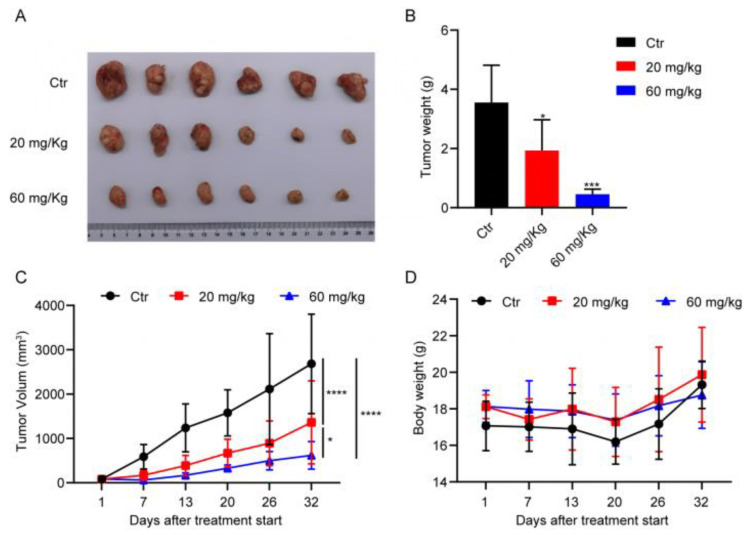
Inhibition of in vivo tumourigenesis in a xenograft mouse model via the oral administration of DHPITO. (**A**) Representative images of nude mice showing HCT116 tumour growth suppression followed by treatment with DHPITO at doses of 20 and 60 mg/kg. (**B**) In vivo tumour growth inhibition curve for DHPITO in nude mice bearing HCT116 xenografts. (**B,C**) Relative tumour volume (mm^3^) over time (days) and tumour weight were measured in HCT116 xenografts following treatment with DHPITO. Mean tumour volumes or weight ± SD are shown (n = 6 mice per group). Tumour volume was calculated according to the following formula: V = 0.5 × W2 × L, where W, width (mm) and L, length (mm). (**D**) The body weight of mice from each group was not significantly influenced by DHPITO treatment, suggesting no major toxicity. All data are shown as the mean ± SD for three independent experiments. * *p* < 0.05; *** and **** *p* < 0.001 versus vehicle.

Given that we were able to ascertain that DHPITO could effectively inhibit microtubular depolymerisation, and promote cell-cycle arrest and apoptosis, the effects of DHPITO on the suppression of tumour formation in vivo were subsequently evaluated. When the xenografted tumours reached a size of approx. 100 mm^3^, the vehicle and DHPITO (20 and 60 mg/kg) were orally administered at 3-day intervals over a period of 32 days. As shown in Figure 6A, tumours formed by HCT116 cells in DHPITO-treated mice were significantly smaller compared with tumours detected in the vehicle-treated control mice. Notably, DHPITO treatment at concentrations of 20 and 60 mg/kg markedly suppressed human CRC tumour growth after 32 days in a dose-dependent manner compared with the vehicle-treated control mice in the xenograft animal model (Figure 6B,C). Importantly, systemic toxicity was likely low during the treatment, since no mortality, clinical sign, or body weight loss was detected for any groups of the modelled mice, as shown in Figure 6D. These results collectively provide convincing pharmacological evidence that DHPITO has potential as an antitumour inhibitor for the treatment of human CRC. 

## 3. Discussion

Microtubules are polymers that form part of the cytoskeleton, and are fundamentally responsible for forming structural scaffolds, cytoplasmic organisation, intracellular transport, and cell division [14]. Microtubules are involved in the formation of the mitotic spindle and form a highly dynamic filament network participating in assembly, dynamic structural maintenance, and disassembly processes for accurate chromosomal congression and segregation during cell division [15]. Therefore, targeting microtubules is an attractive strategy for the treatment of various types of cancer. Although taxanes (such as paclitaxel and docetaxel) have provided a vital therapeutic strategy for decades in terms of treating several different human malignancies, these therapies frequently become ineffective as a consequence of an increasing incidence of drug resistance and mutations in drug targets [16,17]. Therefore, there is an urgent need to discover new generations of tubulin inhibitors that differ entirely from the reported structures of taxanes.

Our research group had developed a novel methodology to construct a diverse suite of 2-disubstituted indolin-3-ones using a unique Ugi/Dieckmann condensation protocol. Moreover, with the same procedure, functional and druglike pyrazino[1,2-a]indoles were also generated through the replacement of normal carbolic acid with bromoacetic acid. Among all the synthesised 2-disubstituted indolin-3-ones and indole-3-one-fused pyrazinos, Compound 6h exhibited the highest level of inhibitory activity against hepatocellular carcinoma cells [10]. Nevertheless, we identified here that DHPITO was the compound that most effectively suppressed the proliferation of CRC cells, and the present results are less consistent with our previous data, perhaps because distinct structural differences might be key to the inconsistent levels of anticancer activity observed with different cancer cell types. Interestingly, our in vitro tubulin polymerisation assay showed that DHPITO could directly prevent the depolymerisation of tubulin and promote the assembly of microtubules, similarly to paclitaxel. However, the structure of DHPITO comprises a pyrazino [1,2a]indole skeleton, thereby completely differing from paclitaxel, which is characterised by the presence of a tetracyclic diterpenoid core, showing that different skeletal structures are responsible for the identical functions of these compounds. Hence, the interaction domain of DHPITO with tubulin may be different from that of the other reported promoters of tubulin polymerisation, and DHPITO may also be able to overcome the persistent drug resistance that is associated with already existing taxanes, and have reduced levels of toxicity. All these possibilities demonstrate that DHPITO is a novel promoter of tubulin polymerisation. However, we did not determine whether DHPITO acts according to the same mechanism in vitro and in vivo, and our future studies will evaluate other health indicators of mice in addition to performing reverse-transcription quantitative polymerase chain reaction (RT-qPCR) and immunoblotting experiments to detect the mRNA and protein expression levels of key proteins of interest, as described by Zhu et al. [18]. 

Microtubule-targeting agents exert their anticancer effects by strongly arresting cells in the G2/M phase [19]. During the M phase, duplicated chromosomes are localised and attached to the mitotic spindle for precise and time-sensitive segregation into the daughter cells [20]. This orchestrated process requires rapid and highly coordinated microtubule dynamics. Stabilising agents of microtubules such as paclitaxel, docetaxel, and etoposide lead to the arrest of the cell cycle at mitosis by causing the kinetic suppression (stabilisation) of microtubule dynamics, which prevents microtubular depolymerisation shrinkage and eventually induces apoptosis [21]. As with other microtubule-targeting agents, cell-cycle analytical experiments performed in the present study revealed that DHPITO arrested CRC cells at the G2/M phase. Consistently with this, protein EB1 accumulated at the cell equator after treatment with DHPITO, whereas the chromosome or chromosomal fragments remained between the two poles, suggesting that the loss of microtubular depolymerisation shrinkage in the presence of DHPITO induced the aggregation of the microtubules at the equatorial plate with the subsequent failure of chromosomes to migrate to the poles of cells. Additionally, apoptosis was activated following exposure to DHPITO, as shown by the cleavage of PARP. The upregulation of p-H3 at Ser10 also demonstrates that apoptosis occurred primarily during the mitotic phase of the cell cycle. Microtubules play an important role in cell motility, in addition to their effects on regulating the progression of mitosis [22,23]. In accordance with these findings, the results of the present study show that the significantly decreased migration and invasion rates were induced in a dose-dependent manner following treatment with DHPITO. Accordingly, the overall effects of DHPITO were similar to those of known microtubule stabilisers, namely, a reduction in cell motility and arrest of the cell cycle at the G2/M phase, eventually resulting in apoptosis. 

In conclusion, the results obtained in the current study present a novel tubulin inhibitor that is able to promote tubulin polymerisation. The main advantage associated with the production of DHPITO is that the synthesis method is simple, efficient, ecofriendly, and cheap. Moreover, among all the synthesised compounds, DHPITO exhibited the most potent inhibitory effects against CRC cells. In vitro studies demonstrated that DHPITO could inhibit cell migration and invasion, and induce cell apoptosis through G2/M phase arrest in both the HCT8 and HCT116 cell lines. Furthermore, DHPITO was very efficacious in terms of its antitumoural activity in HCT116 tumour xenografts in nude mice, with no weight loss or any other obvious adverse effects observed in the model mice. Taken together, the findings in the present study partly uncover the antiproliferative and antitumour mechanisms of DHPITO, providing ample evidence of its potential as a promising new lead compound in the discovery of chemotherapeutic agents.

## 4. Materials and Methods

### 4.1. Reagents and Antibodies

3-(4,5-Dimethylthiazol-2-yl)-2,5-diphenyltetrazolium bromide (MTT) (cat. no. A600799-0005) was purchased from Sangon Biotech (Shanghai, China), and paclitaxel (cat. no. S1150) was purchased from Selleck Chemicals (Houston, TX, USA). Propidium iodide (PI) (cat. no. ST512), 4,6-diamidino-2-phenylindole (DAPI) (cat. no. C1002), and Crystal Violet Staining Solution (cat. no. C0121) were obtained from Beyotime Institute of Biotechnology (Shanghai, China). The Epithelial–Mesenchymal Transition (EMT) Antibody Sampler Kit (cat. no. 9782T), and the anti-p21 (cat. no. 2947S), antiphosphor-histone H3 at Ser10 (cat. no. 53348S), antipoly ADP-ribose polymerase (anti-PARP) (cat. no. 9532S), anticytochrome *c* (cat. no. 4272S), anti-B-cell lymphoma 2 (anti-BCL-2) (cat. no. 15071S), anti-BCL2- associated X protein (anti-Bax) (cat. no. 41162S), anticaspase-8 (cat. no. 9746S), anti-α-tubulin (cat. no. 3873S) and antiglyceraldehyde-3-phosphate dehydrogenase (anti-GAPDH) (cat. no. 5174S) antibodies were purchased from Cell Signaling Technology, Inc. (Danvers, MA, USA) Lastly, antimicrotubular end-binding protein 1 (anti-EB1) (cat no. 17717-1-AP) and anti-p53 (cat no. 10442-1-AP) polyclonal antibodies were purchased from Proteintech Group, Inc. (Chicago, IL, USA).

### 4.2. Cell Culture 

Human CRC cell lines (HCT116, HCT8 and SW480) and fetal human colon cells (FHC) were purchased from the American Type Culture Collection (ATCC). HCT116 cells were cultured in McCoy’s 5A medium (cat no. 16600108; Thermo Fisher Scientific, Inc.) supplemented with 10% fetal bovine serum (FBS) (cat no. 10100147; Thermo Fisher Scientific, Inc.), whereas the HCT8, SW480, and normal adult colonic epithelial line FHC cells were maintained in high-glucose Dulbecco’s Modified Eagle Medium (DMEM) (cat no. SH30022.01, Thermo Fisher Scientific, Inc. (Waltham, MA, USA)) containing 10% FBS. Cells were grown in an incubator in a humidified atmosphere with 5% CO_2_ at 37 °C.

### 4.3. MTT Assay

The effect of the compounds on the viability of the HCT116 and HCT8 cells was determined using an MTT assay. Briefly, HCT116 and HCT8 cells were harvested after having reached 80% confluence, counted, and seeded at a density of 1 × 10^3^ cells per well into a 96-well plate containing 100 μL Complete™ medium. After culture for 24 h, cells were incubated in 100 μL of fresh Complete™ medium supplemented with 10 μM compounds or different concentrations of DHPITO (0, 0.39, 0.78, 1.56, 3.125, 6.25, 12.5, 25, and 50 μM) for 48 h or 1, 2, 3, 4 or 5 days, respectively. Subsequently, 20 μL of MTT solution (5 mg/mL) was added to each well and incubated with the cells for 4 h. The medium was then discarded, and 200 μL dimethylsulfoxide (DMSO) was added to each well to dissolve the formazan. The absorbance was measured at 570 nm using a microplate reader (Bio-Tek Instruments, Inc., Winooski, VT, USA) after shaking the plate on a shaker for 10 min. All experiments were repeated three times, and the cell viability curves of the HCT116 and HCT8 cell lines were calculated using GraphPad^®^ Prism 9.0 software (Dotmatics).

### 4.4. 5-Ethynyl-2’-deoxyuridine (EdU) Incorporation Assay

The effect of DHPITO on the cell proliferation of CRC cells was approximated using a BeyoClick™ EdU Cell Proliferation Kit with Alexa Fluor 594 (cat no. C0078S; Beyotime Institute of Biotechnology) while precisely following the manufacturer’s instructions. Briefly, cells were harvested, counted, seeded into the CellCarrier™-96-well plate (PerkinElmer, Inc., Waltham, USA) at a density of 1 × 10^4^ cells per well, and cultured overnight. Cells were treated with DHPITO for 24 h at concentrations of 0, 10, 20 or 40 μM; subsequently, the cells were incubated with 10 μM EdU working solution for 4 h at 37 °C. After fixation with 4% paraformaldehyde for 30 min at room temperature, the cells were blocked with QuickBlock™ Blocking Buffer for Immunol Staining (cat no. P0260; Beyotime Institute of Biotechnology, Shanghai, China) for 1 h at 37 °C. Thereafter, the cells were incubated in a click reaction buffer for EdU staining for 30 min in a dark environment before being stained with Hoechst 33342 for 10 min. Finally, the images were captured and approximated using an Operetta CLS High Content analytical system (PerkinElmer, Inc., Waltham, MA, USA). 

### 4.5. Colony Formation Assay 

For the colony formation assay, HCT116 and HCT8 cells were seeded into a 6-well plate at a density of 1000 cells per well supplemented with 2 mL of Complete™ medium and incubated for 24 h. After treatment with DHPITO at a concentration of 0, 10, 20, or 40 μM for 10 days, the formed colonies were washed with cold phosphate-buffered saline (PBS), fixed with 4% paraformaldehyde for 30 min, and then stained with 0.5% crystal violet for 30 min at room temperature. The plates were scanned and visualised using a Perfection V800 Photo scanning apparatus (Seiko Epson Corporation, Suwa, Japan). All statistical measurements were performed in triplicate.

### 4.6. Cell-Free Tubulin Polymerisation Assay In Vitro

The in vitro tubulin polymerisation assay was performed as reported previously [24], and a Tubulin Polymerisation Assay Kit (cat no. BK006P; Cytoskeleton, Inc., Denver, CO, USA) was used to measure the effects of DHPITO on microtubular polymerisation. Briefly, the purified tubulin (4 mg/mL) was dissolved in a PEM buffer comprising 80 mM PIPES (P; pH 6.9), 0.5 mM EGTA (E), and 2 mM MgCl_2_ (M), and supplemented with 15% glycerol that had been pretreated with DHPITO (5 or 20 µM), paclitaxel (5 µM), or vehicle (DMSO) at 4 °C. Following the addition of 1 mM GTP, the absorbance kinetics of 61 cycles for each sample was measured at 340 nm and at 37 °C using a microplate reader at an interval of 1 min.

### 4.7. Immunofluorescence Analysis

HCT116 and HCT8 cells were harvested and seeded into the CellCarrier™-96-well plate at a density of 1 × 10^4^ cells per well. HCT116 and HCT8 cells were treated with DHPITO (0, 10, 20, or 40 µM) or paclitaxel (500 nM) for 12 h, fixed with 4% paraformaldehyde for 30 min at room temperature, and then blocked with QuickBlock™ Blocking Buffer containing 0.1% Triton X100 at room temperature for 1 h. The samples were subsequently washed with PBS, followed by incubation with antiphosphor-histone H3 at Ser10, anti-α-tubulin, or anti-EB1 antibodies at 4 °C overnight. Cells were then incubated with the fluorescent goat antirabbit IgG (H + L) Highly Cross secondary antibody (cat no. A32731, Thermo Fisher Scientific, Inc.) at room temperature for 1 h in a dark environment prior to staining with DAPI for 15 min at room temperature. Fluorescence images were captured and approximated using the High Content Analysis System of PerkinElmer, Inc.

### 4.8. Western Blot Analysis

HCT116 and HCT8 cells were treated with the indicated concentrations (0, 10, 20, and 40) of DHPITO for 12 h before being collected and lysed with radioimmunoprecipitation assay (RIPA) buffer (150 mM NaCl, 1% Nonidet P-40 (NP-40), 1% sodium deoxycholate, 0.1% sodium dodecyl sulfate (SDS), 25 mM Tris-HCl (pH 7.4) and 1 mM EDTA (pH 8.0)) supplemented with protease and phosphatase inhibitors (cat no. 5892791001; Roche Diagnostics, Mannheim, Germany) on ice. The lysates were centrifuged at 12,000× *g* for 20 min, and the pellets and supernatants were collected to detect the assembled (cytoskeletal) and unassembled (cytosolic) forms of tubulin. Subsequently, the protein concentration of the supernatants was measured using a bicinchoninic acid (BCA) protein assay kit (cat no. P0010; Beyotime Institute of Biotechnology). A total of 50 μL 1× loading buffer was added to the pellets, which were then boiled for 10 min. An equal amount of each sample (30 μg) was loaded onto 10% and 15% SDS-polyacrylamide gel electrophoresis (PAGE) gels, followed by transfer to polyvinylidene difluoride (PVDF) membranes (MilliporeSigma). The membranes were blocked with nonfat dry milk (cat no. 9999S; Cell Signaling Technology, Inc., Danvers, MA, USA) for 1 h at room temperature; afterwards, they were incubated with the respective primary antibodies as referenced in the Reagents and Antibodies section above at 4 °C overnight, and subsequently incubated with IRDye^®^ 800CW goat antimouse IgG (H + L) or IRDye^®^ 680LT donkey antirabbit IgG (H + L) (LI-COR Biosciences, Lincoln, NE, USA) secondary antibody at room temperature in a dark environment for 1 h. Lastly, immunoreactivity was visualised using an Odyssey Two-Color Infrared fluorescence Imaging System (LI-COR Biosciences). 

### 4.9. Flow Cytometric Analysis

For cell-cycle analysis, 5 × 10^5^ cells per well were plated in 60 mm dishes and cultured for 24 h at 37 °C prior to treatment with DHPITO at a concentration of 0, 10, 20, or 40 μM for 24 h. Cells were collected with trypsinisation and fixed in cold 70% ethanol overnight at 4 °C. After washing with PBS, the cells were incubated with PBS supplemented with PI (cat. no. ST512; Beyotime Institute of Biotechnology) and Rnase A (cat no. ST578; Beyotime Institute of Biotechnology) for 30 min at 37 °C, and approximated using a BD Accuri™ C6 Flow Cytometer interfaced with Cell Quest software (BD Biosciences). The cell-cycle distribution was approximated using FlowJo 7.6.1 analytical software (BD Biosciences, Franklin Lakes, NJ, USA). For apoptotic analysis, HCT116 and HCT8 cells were seeded at a density of 5 × 10^5^ cells per well in a 60 mm dish and treated with DHPITO at the indicated concentrations (0, 10, 20, and 40 μM) for 24 h. After washing with PBS, the cells were trypsinised, harvested, and simultaneously stained with annexin V-fluorescein isothiocyanate (FITC) and PI using an Annexin V-FITC Apoptosis Detection Kit (cat no. C1062M; Beyotime Institute of Biotechnology). Lastly, the samples were subjected to flow cytometric analysis, and the apoptotic ratio was approximated using FlowJo 7.6.1 analytical software.

### 4.10. Wound-Healing Assay

A wound-healing assay was performed using an Ibidi cell culture insert (cat no. 80206; Ibidi GmbH) to measure cell migration according to the manufacturer’s instructions. Briefly, HCT116 and HCT8 cells were harvested, seeded into the culture insert at a density of 1 × 10^5^ cells per culture insert, and allowed to grow until they had reached 95% confluence. Subsequently, the culture insert was taken away, and cells were further treated with DHPITO for either 24 or 48 h at concentrations of 0, 10, 20, and 40 μM. The wound gap was photographed using an inverted-phase contrast microscope (Olympus Corporation) at 0, 24, and 48 h. Lastly, the wound-healing area was measured using Image J software (downloaded from https://imagej.nih.gov/ij/download.html, accessed on 1 January 2023; NIH), and the wound-healing rate was calculated according to the following formula: wound healing rate = (initial wound area − residual wound area)/initial wound area × 100%.

### 4.11. Transwell Invasion Assay

The effect of DHPITO on cell invasion was evaluated using a Transwell invasion assay as previously described [25]. Briefly, a 24-well Transwell chamber with 8 μm pore size (Corning, Inc.) was used to perform the assay. HCT116 and HCT8 cells were harvested, resuspended in 200 µL McCoy’s 5A and DMEM media without serum containing 0, 10, 20, or 40 μM DHPITO, and then seeded into the upper chamber of the wells. Complete™ medium (500 µL) was added to the lower chamber. After incubating the cells for 48 h, they were then fixed with 4% paraformaldehyde at room temperature for 30 min and stained with Crystal Violet Staining Solution (cat no. C0121; Beyotime Institute of Biotechnology). The numbers of migrated cells were counted under an inverted phase-contrast microscope (Olympus Corporation, Tokyo, Japan) microscope. 

### 4.12. In Vivo Mouse Xenograft Model

All the animal studies performed in the present study were reviewed and approved by the Ethics Committee for Animal Studies at Chongqing University of Arts and Sciences, Chongqing, China. Nude Balb/c mice were purchased from Hunan SJA Laboratory Animal Co., Ltd. (Hunan, China). All experimental procedures were performed while the animals were under 2.5% isoflurane gas anaesthesia. 

The in vivo xenograft model was set up as previously described [26]. Briefly, nude Balb/c mice (female, aged 4–6 weeks, weighing approx. 20 g) were inoculated subcutaneously on their flanks with 1 × 10^6^ HCT116 cells suspended in 100 μL serum-free McCoy’s 5a medium. After the mean tumour volume had reached approx. 100 mm^3^, the mice were randomly divided into 3 groups (n = 6 per group) and orally administered every 3 days with the vehicle, 20 or 60 mg/kg DHPITO dissolved in 100 μL solvent containing 5% DMSO, 30% polyethylene glycol 300 (PEG300), 10% Tween-80, and 55% saline. The tumour lengths (L) and widths (W) were measured using a vernier calliper every 3 days, and the tumour volume was calculated according to the following formula: V (volume) = (L × W^2^)/2. After 32 days of treatment, the mice were euthanised by cervical dislocation, and tumours were dissected and weighed.

### 4.13. Statistical Analysis 

All experiments were performed in triplicate. GraphPad^®^ Prism version 9.0 (Dotmatics) was used for statistical analysis. Data are presented as the mean ± standard deviation, and analysis of variance (ANOVA) was used to compare differences between groups. *p* < 0.05 was considered a statistically significant value. 

## Data Availability

The datasets used and/or analysed during the current study are available from the corresponding author on reasonable request.

## Supplementary Materials

The following supporting information can be downloaded at: https://www.mdpi.com/article/10.3390/molecules28041948/s1, Figure S1: Cytotoxicity of the synthesized derivatives in HCT116 cells; Figure S2: The concrete morphology of HCT8, HCT116 and FHC cell lines after treatment with DHPITO were detected by microscopic analysis; Figure S3: Colony formation assay was conducted using FHC cells to evaluate inhibitory growth in vitro after treatment with DMSO, 10, 20 and 40 μM DHPITO for 10 days; Figure S4: DHPITO and paclitaxel acts as microtubule polymerization stabilizers; Figure S5: DHPITO is capable of increasing intracellular EB1 accumulation; Figure S6: Treatment with DHPITO does not induce FHC cells to undergo apoptosis.
