# Supplementary material for: Pyrrolyldihydropyrazino[1,2-a]indoletrione Analogue Microtubule Inhibitor Induces Cell-Cycle Arrest and Apoptosis in Colorectal Cancer Cells
Source: Molecules. 2023 Feb 17;28(4):1948. doi: 10.3390/molecules28041948 (PMC9966721; doi:10.3390/molecules28041948)
Supplement: Supplementary file 1 [file molecules-28-01948-s001.zip › molecules-2162858-supplementary.pdf]

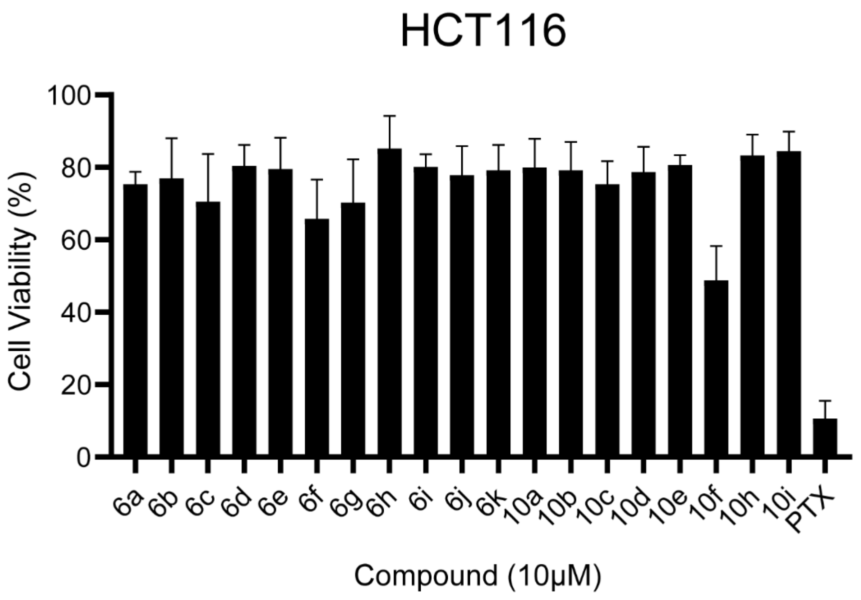

Figure S1. Cytotoxicity of the synthesized derivatives in HCT116 cells. The *in vitro* proliferation of HCT116 cells after treatment with indicated compounds was evaluated by the MTT assay.

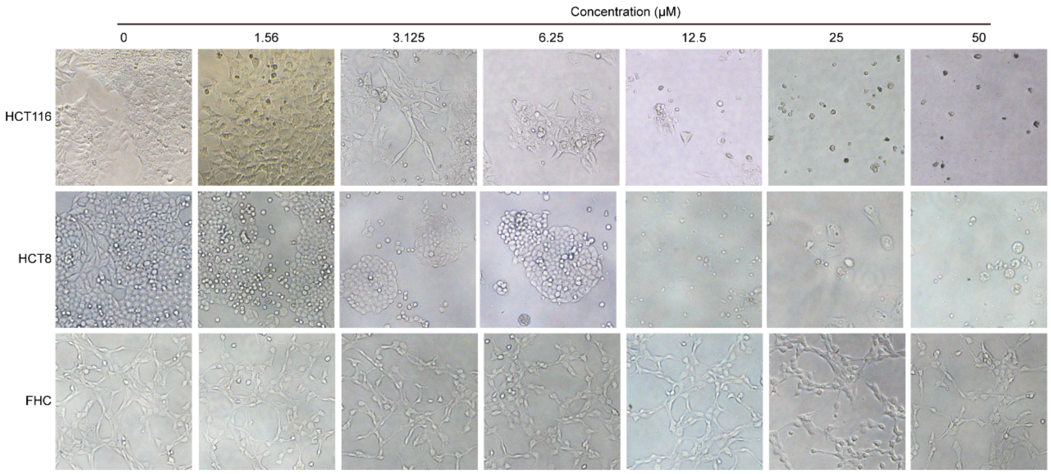

Figure S2. The concrete morphology of HCT8, HCT116 and FHC cell lines after treatment with DHPITO were detected by microscopic analysis. HCT8, HCT116 and FHC cell lines were plated into 96-well plates and treated with the indicated concentrations of DHPITO for 5 days. Subsequently, the cell morphology was observed under a light microscope.

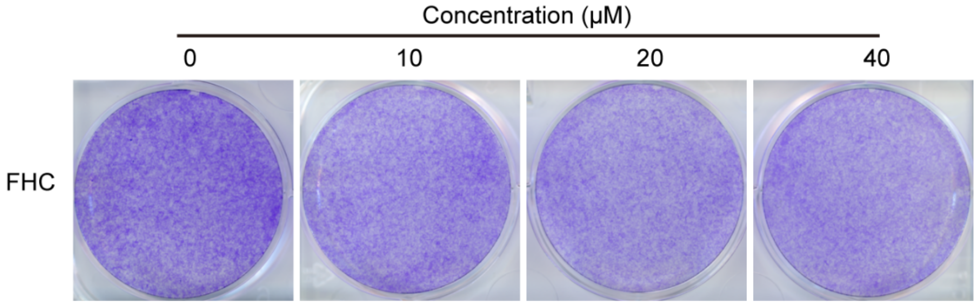

Figure S3. Colony formation assay was conducted using FHC cells to evaluate inhibitory growth *in vitro* after treatment with DMSO, 10, 20 and 40 μM DHPITO for 10 days. The colonies were visualized by staining with crystal violet blue.

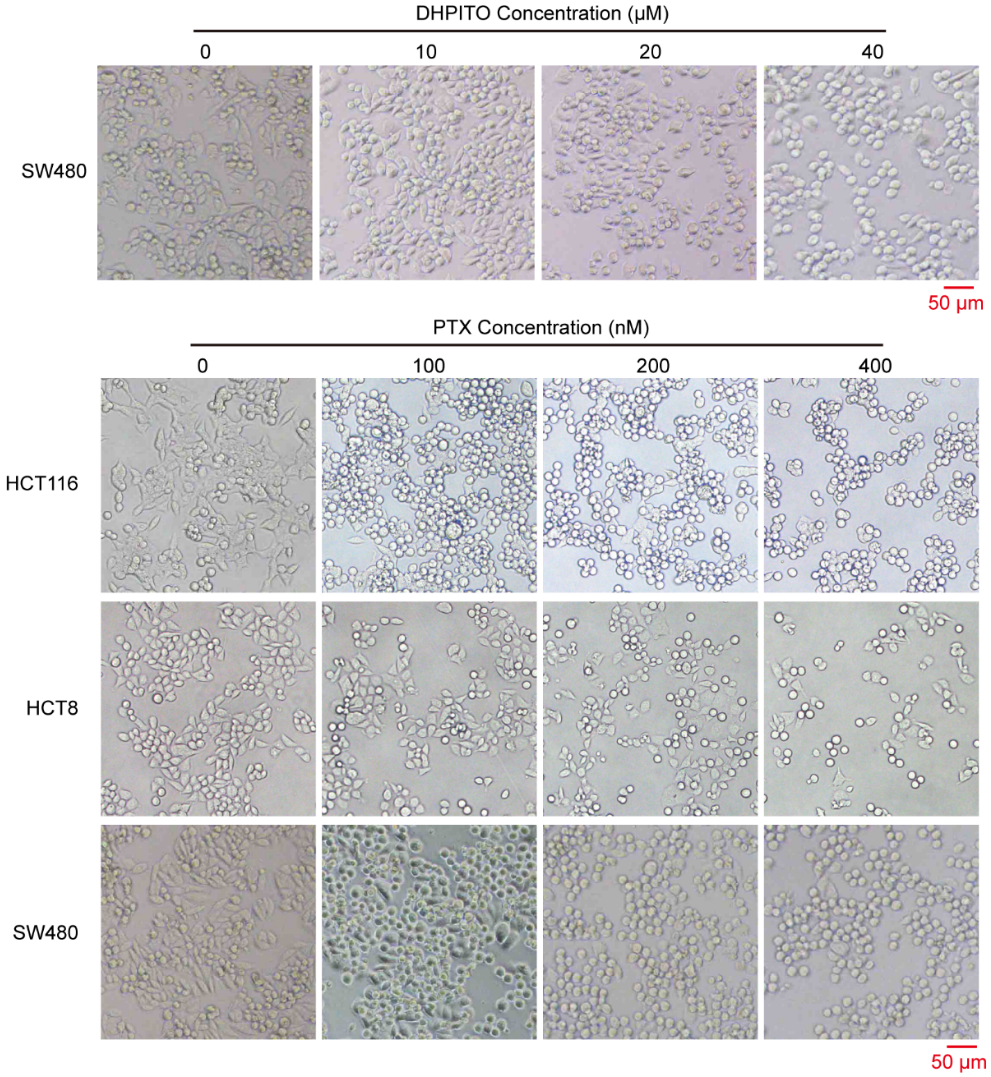

Figure S4. DHPITO and paclitaxel acts as microtubule polymerization stabilizers. Cell morphology was observed under a phase-contrast microscope in the presence of

the indicated concentrations of DHPITO or paclitaxel. Paclitaxel was used in the assay as a known enhancer of tubulin polymerization. Scale bar, 50 μm.

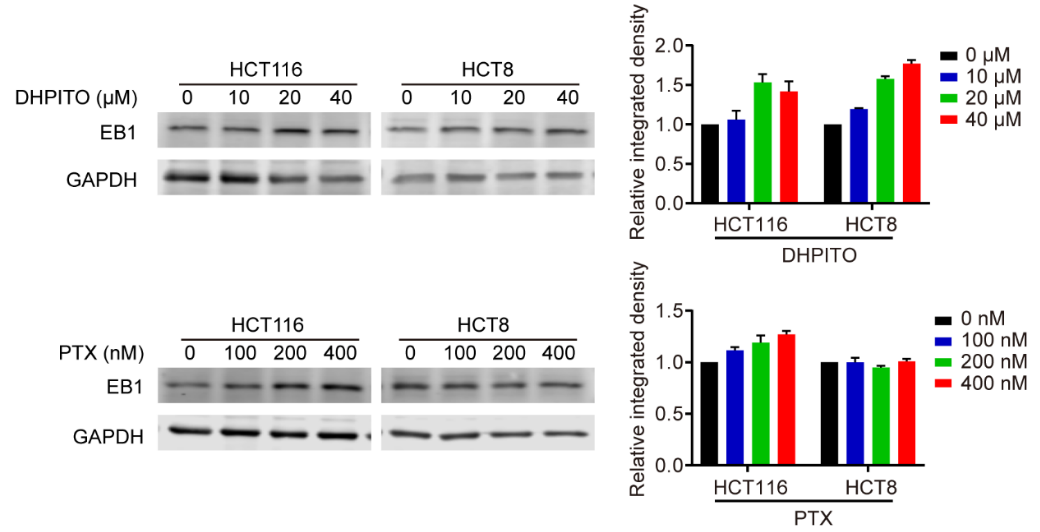

Figure S5. DHPITO is capable of increasing intracellular EB1 accumulation. Western blotting was employed to assess the EB1 protein levels in both HCT116 and HCT8 cells after exposure to the indicated doses of DHPITO and paclitaxel. GAPDH was used as a loading control. All data were shown as the mean ± SD for three independent experiments. *P<0.05; **P<0.01; *** and **** P<0.001, and 'ns' represents no significant difference versus vehicle. EB1, microtubule end-binding protein 1.

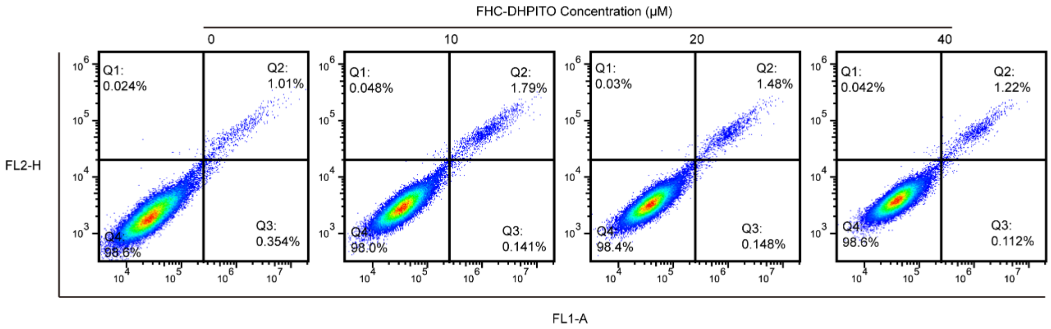

Figure S6. Treatment with DHPITO does not induce FHC cells to undergo apoptosis. Flow cytometry analysis using annexin V/PI staining was used to detect the cell apoptosis after treatment with indicated concentrations of DHPITO in FHC cells. PI, propidium iodide.
